# Mr. William R. Warner

**Published:** 1901-05

**Authors:** 


Mr. William R. Warner, of Philadelphia, the founder of the firm of Wm. R. Warner & Co., manufacturing chemists, died at his home in that city Wednesday, April 3, 1901. He was distantly related to George Washington, and his art collection included over 100 portraits of the Father of his Country, as well as portraits of distinguished physicians, scientists and pharmaceutists. In 1860 he was appointed a member of the committee of revision of the United States Pharmacopeia, and he was for many years a member of the Philadelphia College of Pharmacy.

